# Peripheral and gastrointestinal immune systems of healthy cattle raised outdoors at pasture or indoors on a concentrate-based ration

**DOI:** 10.1186/1746-6148-6-19

**Published:** 2010-03-31

**Authors:** Alexandre Lejeune, Frank J Monahan, Aidan P Moloney, Bernadette Earley, Alistair D Black, Deirdre P Campion, Tanya Englishby, Petrina Reilly, John O'Doherty, Torres Sweeney

**Affiliations:** 1 UCD School of Agriculture, Food Science and Veterinary Medicine, University College Dublin, Belfield, Dublin 4, Ireland; 2 Teagasc, Grange Beef Research Centre, Dunsany, Co. Meath, Ireland

## Abstract

**Background:**

Despite an increasing preference of consumers for beef produced from more extensive pasture-based production systems and potential human health benefits from the consumption of such beef, data regarding the health status of animals raised on pasture are limited. The objective of this study was to characterise specific aspects of the bovine peripheral and the gastrointestinal muscosal immune systems of cattle raised on an outdoor pasture system in comparison to animals raised on a conventional intensive indoor concentrate-based system.

**Results:**

A number of *in vitro *functional tests of immune cells suggested subtle differences between the animals on the outdoor versus indoor production systems. There was a decrease in the number of neutrophils and monocytes engaged in phagocytosis in outdoor cattle (*P *< 0.01 and *P *< 0.05, respectively) in comparison to those indoors. Following mitogen stimulation, a lower level of interferon-γ was produced in leukocytes from the outdoor animals (*P *< 0.05). There was evidence of a gastrointestinal nematode infection in the outdoor animals with elevated levels of serum pepsinogen (*P *< 0.001), a higher number of eosinophils (*P *< 0.05) and a higher level of interleukin-4 and stem cell factor mRNA expression (*P *< 0.05) in the outdoor animals in comparison to the indoor animals. Lower levels of copper and iodine were measured in the outdoor animals in comparison to indoor animals (*P *< 0.001).

**Conclusion:**

Despite distinctly contrasting production systems, only subtle differences were identified in the peripheral immune parameters measured between cattle raised at pasture in comparison to animals raised on a conventional intensive indoor concentrate-based production system.

## Background

Emerging data suggest that there are human health advantages associated with the consumption of grass-fed beef compared with beef produced from intensive concentrate-based systems [1]. There is also some evidence that regular outdoor exercise has positive effects on the health status of cattle [2,3]. The outdoor environment may expose animals to different immune challenges in comparison to the indoor environment, but whether this leads to a differentially activated immune system has not been fully investigated. Indeed, only one research group has examined the effect of outdoor versus indoor housing on immune status and this study was performed on pigs [4]. These authors identified a lower white blood cell and lymphocyte count, accompanied by a lower natural killer cell activity and a higher neutrophil percentage in outdoor compared to indoor pigs. The authors explained these results by a possible more diluted and thus lower microbial exposure in the outdoor environment.

Among the other potential benefits, outdoor beef production systems could help to reduce the need for antibiotics used as therapeutics and prophylactics in livestock production, and thereby decrease the risk of bacterial antibiotic resistance [5]. Furthermore, it has been suggested that both the energy required to mount an immune response and subsequent depression of appetite can affect nutrient partitioning and therefore lead to production losses [6]. However, the extent to which variation in animal husbandry systems affects animal health and, in particular, characteristics of the bovine immune system, remains an essential important question that has not been comprehensively studied to-date.

Several animal husbandry parameters such as nutrition or space allowance are known to influence health status. For example, the number of white blood cells and lymphocytes in cattle increased slightly during the dry period and early lactation when dairy cows were fed diets that provided a high energy allowance [7]. Among the subpopulation of lymphocytes, an increase in the proportion of B-cells was shown in cows fed with low or high, but not medium, energy diets [7]. A higher lymphocyte count was also identified in grazing beef cattle fed with a corn-based supplement compared to a wheat-based supplement [8]. These authors also identified that the proliferative response of lymphocytes differed according to diet with a corn-based supplement boosting the proliferative response, which could thereby decrease susceptibility to disease in steers.

The objective of this study was to characterise specific aspects of the bovine peripheral immune system and the gastrointestinal muscosal immune system of healthy animals raised either outdoors on grass or indoors on a concentrate-based diet.

## Methods

### Animals

Charolais × Limousin crossbred heifers were randomly assigned at 8 months of age in November 2006 to one of two production systems: outdoor (n = 15) or indoor (n = 15). Animals were maintained on these treatments for 12 months and blood samples were collected in July 2007 (summer) and October/November 2007 (winter).

Heifers on the outdoor production system were maintained outdoors for the duration of the experiment and grazed a predominantly perennial ryegrass pasture. The daily ration was a target herbage dry matter (DM) intake of 0.02 of live weight per heifer above a residual or post-grazing herbage mass of 900 kg DM ha^-1^. The daily allowance was provided by adjusting the grazing area based on an estimate of grass DM yield/ha.

Heifers in the indoor production system were housed in a slatted floor shed. A concentrate diet (rolled barley, soybean meal, molasses and a mineral/vitamin premix) was offered once daily at an allowance calculated to ensure a similar growth rate to the heifers at pasture. These heifers were offered straw *ad libitum*. All animals were injected with Ivermection (Qualimec, Janssen Animal Health) and nitroxynil (Trodax, Merial) in November 2006 at the commencement of the treatment regimens. Outdoor cattle were further treated with Doramectin (Dectomax, Pfizer Animal Health) in August 2007.

All procedures described in this experiment were conducted under experimental licence from the Irish Department of Health and Children in accordance with the Cruelty to Animals Act 1876 and the European Communities (Amendments of the Cruelty to Animals Act, 1976) Regulations, 1994 and with the approval of Teagasc, the Irish Agricultural and Food Development Authority.

### Blood collection

Blood samples were obtained by jugular venipuncture. Blood (10 mL) was collected into vacutainer tubes (Cruinn Diagnostics) containing heparin for subsequent harvesting of plasma for the assay of pepsinogen and metabolic variables. An additional 6 mL of blood was collected into vacutainer tubes containing K_3_-EDTA for determination of total and differential leukocyte numbers.

### Haematology

Total circulating leukocyte number and subsets: neutrophil, lymphocyte, monocyte, eosinophil and basophil number were determined from whole blood using an automatic hematology analyzer (AV ADIVA 2120, Bayer Healthcare, Siemens) equipped with software for bovine samples.

### Plasma mineral and pepsinogen determination

Plasma I, Zn and Cu were measurements were carried out at a comemercial analytical laboratory (IODOLAB, 1 Avenue Bourgelat, 69280 Marcy L'étoile, France). Plasma was harvested following centrifugation of whole blood at 1600 × g at 4°C for 15 min and stored at -80°C until assayed. Plasma pepsinogen was determined using the method of Hirschowitz [9] and Ross *et al. *[10].

### Stimulated leukocyte production of interferon-γ

The stimulated production of interferon-γ (IFNγ) by leukocytes was determined following a modification [11,12] to a previously described procedure [13]. Briefly, duplicate 1.5 mL aliquots of blood from 10 animals in each group were cultured in 24 well plates with 20 μL of phosphate buffer saline (PBS) containing 1.0 mg/mL of concanavalin A (Con A, Sigma) mitogen or no additive, for 24 h at 37°C in an atmosphere of 5% CO_2 _in air. The plates were then centrifuged at 1600 × g at 4°C for 20 min and the supernatant harvested and frozen at -20°C until assayed. Production of IFNγ was determined using an ELISA procedure (Bovigam, Biocor Animal Health) as previously described [14].

### Proliferation assay

Peripheral blood mononuclear cells (PBMC) were isolated from 10 animals in each group on a Ficoll-sodium diatrizoate gradient with a specific density of 1.077 g/ml (Histopaque, Sigma) according to the manufacturer's recommendations. The PBMC were suspended in RPMI-1640 (Gibco) containing non-essential amino acids (Sigma), 5% fetal calf serum (Gibco) and gentamycin (50 μg/ml).

Lymphocytes (2 × 10^5 ^cells) were placed in triplicate into a sterile 96-well flat-bottom plate. The mitogen Con A was added at 5 μg/ml and PBS was used as a negative control. After 48 h incubation at 37°C under 5% CO_2_, 3.75 μCi [^3^H]-thymidine (Sigma) was added to each well. Plates were incubated for 24 h and cells were harvested onto a glass filter. The incorporated [^3^H]-thymidine was measured on a Wallac MicroBeta reader. Stimulation index (SI) is defined as the mean CPM of the response of the antigen-stimulated cells divided by the mean of the response of cells cultured without antigen (PBS).

### Phagocytosis assay

The phagotest kit (Orpegen) which measures the uptake of opsonized, FITC-labeled *E. coli *was used to quantity *in vitro *phagocytosis activity according to the manufacturer's protocol. Data were collected from 10,000 cells per sample by flow cytometry (Dako CyanADP) using Summit 4.2 software. The results are reported as the percentage of phagocytosing cells in the monocyte, neutrophil and eosinophil gates.

### Histology

At the end of the study, animals were sacrificed at a commercial abattoir (Meadow Meats Ltd., Rathdowney, Co. Laois, Ireland). Within 20 min of slaughter, the animals were eviscerated, and a sample of approximately 2 cm × 3 cm was taken from the fundic region of the abomasum (6 animals in each group), and placed immediately in 10% buffered formalin (pH 7.4). After 24 h, the samples were processed using an automated processor (Tissue-TEK VIP, Sakura Finetek) and embedded in paraffin wax. Sections (5 μm) were cut using a microtome (Leitz 1512), and these were mounted onto glass slides.

A haematoxylin and eosin stain was used to identify tissue architecture. To specifically identify tissue eosinophils, further slides were stained using Giemsa stain [15]. Eosinophil granules were stained a strong pink colour using this stain. Tissue mast cells were identified using a uranyl nitrate metachromatic method, incorporating a toludine blue staining step [16]. The mast cell granules were stained a distinct dark blue using this method.

Image-Pro Plus software (Version 5, Media Cybernetics), was used as a stereological tool in all histological studies described. To determine cell numbers, images were gathered using a colour video camera attached to a microscope linked to the image analysis programme, which allowed measurement of the area being examined. Eosinophils were enumerated under 400 × magnification. A thresholding technique was used to aid counting of eosinophils. To achieve this, the image analysis programme was set to select areas of specific eosinophil granule colour. Collections of granules less than 3 μm in diameter were excluded from the count. The larger mast cells were enumerated under 200 × magnification. To identify the primary location of the cells, the mucosal area was divided into two regions: the upper mucosa, comprising the lumen to the base of the gastric pits, and the lower region, comprising the glandular areas of the mucosa. Eosinophil and mast cell counts were based on up to 10 random fields in each region in each slide. Results were presented as cell count per mm^2^.

### Total RNA extraction and cDNA synthesis

Total RNA from samples of the small intestine was extracted using TRI reagent (Sigma) and Tissue Lyzer (Qiagen) according to the manufacturer's protocols (10 animals were used in each group). The extracted RNA was treated with DNase I (Qiagen) at room temperature for 10 min to remove contaminating genomic DNA. The quality of the total RNA was verified on a 1% agarose gel and the amount and purity was determined with a NanoDrop ND-1000 spectrophotometer (NanoDrop Technologies). Random hexamer primed cDNA synthesis was performed using 1 μg of total RNA and SuperScript III Reverse Transcriptase (Invitrogen) in a final volume of 20 μL according to manufacturer's recommendations.

### Real-time PCR

Each reaction was carried out in a 10 μL reaction mixture containing 1 μL cDNA, 5 μL Power SyBr Green PCR master mix (Applied Biosystems) and primers at a final concentation of 300 nM. Interferon-γ, interleukin 10 (IL-10) and tumor necrosis factor alpha (TNFα) primers were as previously described [17]. Tumor necrosis factor beta (TNFβ), granulocyte macrophage colony stimulating factor (GM-CSF), interleukin 4 (IL-4), interleukin 5 (IL-5), interleukin 13 (IL-13) and stem cell factor (SCF) primers were designed using Primer Express 3.0 software (Applied Biosystems) (Table 1). Primers for the reference genes glyceraldehyde-3-phosphate dehydrogenase (GAPDH), acidic ribosomal protein large (RPLP0) and beta-actin (β-Actin) were as previously described [18]. Real-time PCR was carried out using an ABI 7300 real-time PCR system (Applied Biosystems) with the following amplification condition: 50°C for 2 min, 95°C for 10 min, 40 cycles at 95°C for 15 s and 60°C for 1 min. Raw Ct values were transformed to relative quantities using the delta-Ct formula *Q *= *2*^ΔCt ^where Δ^Ct ^is the sample with the highest expression (minimum Ct value) from the data set minus the Ct value of the sample in question. Data from reference genes were then analysed using geNORM, resulting in GAPDH and β-Actin being identified as the 2 most stable genes (M value = 0.473) [19]. A normalization factor was calculated for each sample as the geometric mean of these 2 genes, and used to normalize cytokine gene expression data.

**Table 1:** Sequences of primers used for qRT-PCR

Gene symbol	Gene name	Primer sequence (5'-3')
TNFβ	Tumor necrosis factor beta	CCTCAGCCCAGTAGTGTCTTC CCTATTTGTTTCTTTCTGGATGTTTCT

GM-CSF	Granulocyte macrophage colony stimulating factor	TGCAGGGCAGCCTCACTAG TCGTAGTGGGTGGCCATCAT

IL4	Interleukin 4	GCCACACGTGCTTGAACAAA TGCCAAGCTGTTGAGATTCCT

IL5	Interleukin 5	TGGTGGCAGAGACCTTGACA TTCCCATCACCTATCAGCAGAGT

IL13	Interleukin 13	CCTGACGAGCAGCATGTACTGT TGCAGTTGGAGATGCTGATCA

SCF	Stem Cell Factor	CCGTAGCATTGCCAGCATT TCCAGTAAAAGGCCCCAAAA

### Statistical analysis

All data, except that for gene expression, were analyzed using the MIXED covariance procedure of SAS (SAS system 8). The statistical model used included the effects of production system, season or tissue localization and the interactions between them. All data were checked initially for normality using the PROC Univariate procedure in SAS. Data were transformed prior to analysis to achieve normal distribution. Animal and error were random effects, and treatment, season, and treatment × season were fixed effects. The difference between means of gene expression values was analyzed by Student's t-test.

## Results

### Animal growth

The objective of having both groups animals grow at similar growth rates was achieved. From mean initial weights of 275.5 and 273.0 kg (s.e.d 2.49, P > 0.05), final liveweights of 512.7 and 506.1 kg (s.e.d. 7.63. P > 0.05) were obtained for the outdoor and indoor animals, respectively.

### White blood cell differential counts

The total number of white blood cells did not differ between outdoor and indoor animals (Table 2). There were no significant differences in lymphocyte and neutrophil numbers between the two groups, and all haematological values were found to be within the normal physiological ranges [20]. For eosinophils an interaction (P < 0.05) was observed with higher numbers in indoor compared to outdoor animals in summer but lower numbers in indoor compared to outdoor animals in winter (P < 0.05).

**Table 2:** Total and differential leukocyte counts.

Item	Reference range	Summer		Winter		*P *value		
		**Outdoor**	**Indoor**	**Outdoor**	**Indoor**	**Production system, P**	**Season, S**	**P × S**

WBC (×10^9^/L)	4.00 - 12.00	10.41 ± 0.50	9.91 ± 0.53	10.67 ± 0.50	10.01 ± 0.50	0.416	0.712	0.726

Lymphocytes (×10^9^/L)	2.50 - 7.50	6.04 ± 0.17	5.82 ± 0.16	6.17 ± 0.17	5.75 ± 0.16	0.914	0.617	0.725

Neutrophils (×10^9^/L)	0.60 - 4.00	3.79 ± 0.16	3.53 ± 0.15	3.63 ± 0.17	3.56 ± 0.15	0.742	0.491	0.518

Eosinophils (×10^9^/L)	0.10 - 1.70	0.46 ± 0.04	0.56 ± 0.04	0.72 ± 0.04	0.63 ± 0.04	0.035	0.464	0.030

Total and differential leukocyte counts from indoor and outdoor animals during summer and winter. Values are depicted as mean ± SEM. P value for the effects of the production system (P), season (S) and production system × season (P × S) interaction are indicated. Physiological reference ranges were indicated according to Knowles *et al. *[20].

### Blood cell counts

There was an interaction (P < 0.05) between production system and season for blood cell counts (Table 3). While there was no difference between production systems in summer, in winter, red blood cell numbers were higher (P < 0.05) for indoor cattle. Hemoglobin values were greater in indoor cattle throughout the experiment (P < 0.001). Similar to the erythrocyte number, a greater difference in hematocrit percentage was observed between the two groups in winter than in summer resulting in a significant production system × season interaction (P < 0.05).

**Table 3:** Haematological measurements.

Item	Reference range	Summer		Winter		*P *value		
		**Outdoor**	**Indoor**	**Outdoor**	**Indoor**	**Production system, P**	**Season, S**	**P × S**

Red Blood Cells (×10^6^/μL)	5 - 10	9.38 ± 0.23	9.53 ± 0.14	8.24 ± 0.13	9.15 ± 0.21	0.022	< 0.001	0.012

Haemoglobin (g/dL)	8 - 15	11.65 ± 0.19	12.98 ± 0.17	11.14 ± 0.26	12.98 ± 0.27	<0.001	0.145	0.145

Haematocrit (%)	24 - 46	37.76 ± 0.66	39.85 ± 0.52	34.99 ± 0.79	40.43 ± 0.66	< 0.001	0.043	0.003

Haematological parameters in indoor and outdoor cattle during summer and winter. Values are depicted as mean ± SEM. P value for the effects of the production system (P), season (S) and production system × season (P × S) interaction are indicated. Physiological reference ranges have previously been determined [53].

### Concentrations of minerals and pepsinogen in blood

There were interactions between production system and season for iodine (P = 0.055), zinc (P < 0.05) and copper concentrations (P < 0.001) (Table 4). Thus, outdoor animals had lower levels of iodine compared to indoor animals both in summer and winter but the difference was greatest in summer. While there was no difference in zinc concentration between the two groups in the summer, in winter, zinc concentration was higher for the indoor group. For blood copper, outdoor cattle had lower levels compared to indoor animals both in summer and winter but the difference was greatest in winter.

**Table 4:** Blood mineral concentrations.

Item	Summer		Winter		*P *value		
	**Outdoor**	**Indoor**	**Outdoor**	**Indoor**	**Production system, P**	**Season, S**	**P × S**

I (μg/L)	20.11 ± 2.16	329.27 ± 27.78	15.00 ± 1.67	254.18 ± 11.26	< 0.001	0.024	0.055

Zn (μmol/L)	13.85 ± 0.79	14.33 ± 0.82	15.38 ± 0.91	18.24 ± 0.69	0.135	< 0.001	0.011

Cu (μmol/L)	3.64 ± 0.68	13.47 ± 0.64	1.55 ± 0.44	15.71 ± 0.69	< 0.001	0.512	< 0.001

Blood concentrations of iodine, zinc and copper in indoor and outdoor cattle during summer and winter. Values are depicted as mean ± SEM. *P *value for the effects of the production system (P), season (S) and production system × season (P × S) interaction are indicated.

There was an interaction (P < 0.05) between production system and season for plasma pepsinogen concentrations. Plasma pepsinogen concentrations were higher in outdoor animals compared to indoor animals in summer (1.61 ± 0.26 *vs *0.53 ± 0.07 U/L) and winter (0.99 ± 0.14 *vs *0.37 ± 0.05 U/L) but the difference between production systems was greatest in summer.

### Phagocytic activity of neutrophils and monocytes

**Figure 1:**
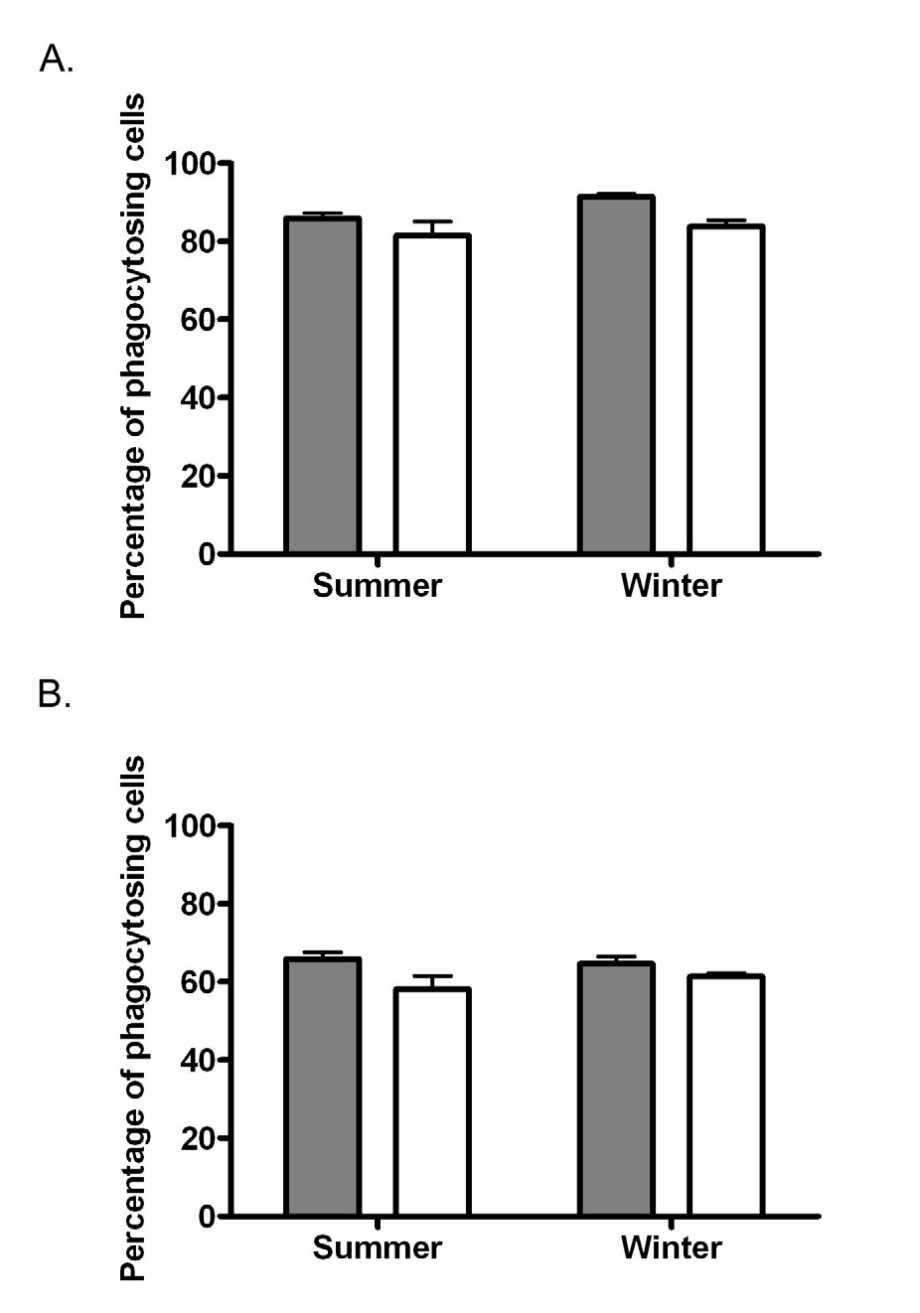
**Phagocytosing neutrophils and monocytes in indoor and outdoor animals**. Percentage of phagocytosing neutrophils (Figure 1A) and monocytes (Figure 1B) in indoor (shaded bars) and outdoor (clear bars) animals during summer and winter. Values are depicted as mean ± SEM. P values for the treatment, season and treatment × season effects were 0.001, 0.033, 0.397, respectively, for neutrophils and 0.021, 0.652, 0.313, respectively, for monocytes.

The percentage of neutrophils and monocytes engaged in phagocytosis was lower (P < 0.05) in outdoor animals compared to indoor animals in both summer and winter (Figure 1A and 1B). Overall, there was a slightly higher (P < 0.05) percentage of phagocytosing neutrophils in winter compared to summer in both groups.

### Interferon gamma production by leukocytes

**Figure 2:**
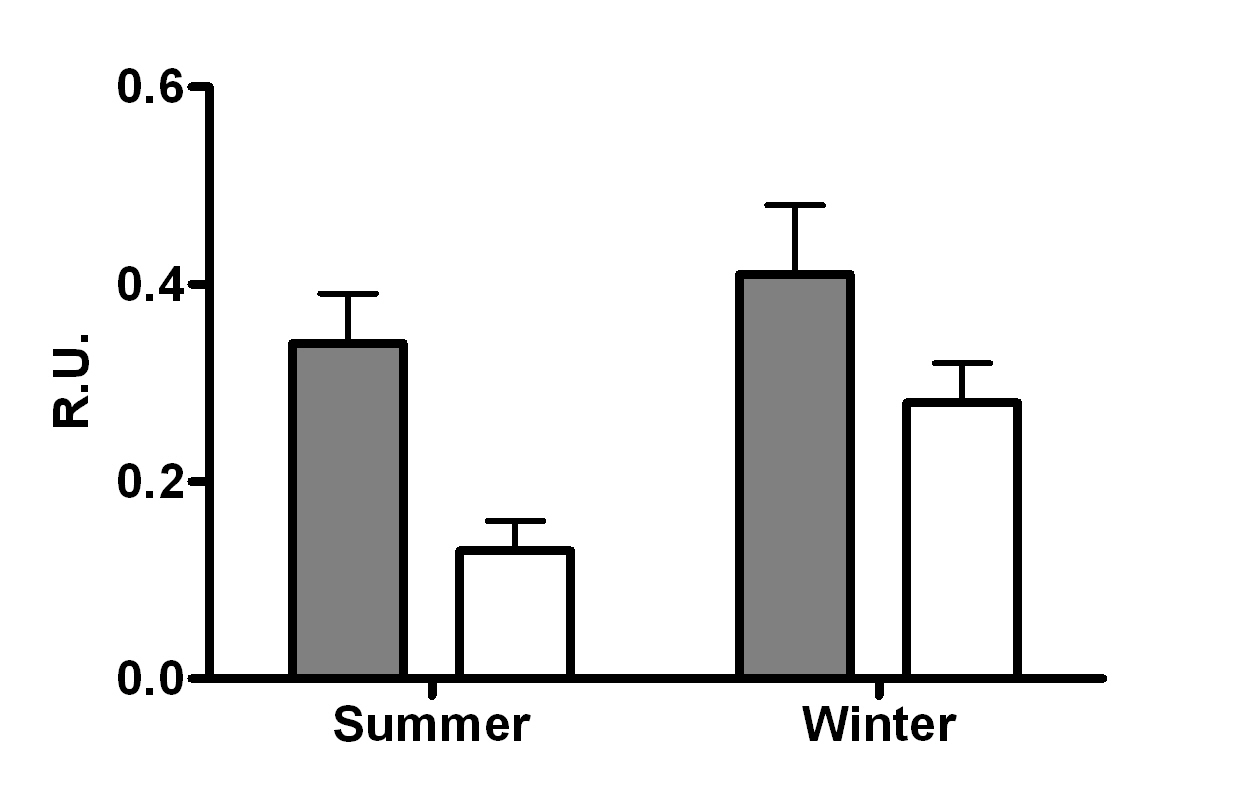
**Con A induced *in vitro *IFN-γ production from cultured leukocytes**. Con A induced *in vitro *IFN-γ production from cultured leukocytes from indoor (shaded bars) and outdoor (clear bars) cattle during summer and winter. Values are depicted as mean ± SEM. P values for the treatment, season and treatment × season effects were 0.011, 0.023, 0.382, respectively.

Following stimulation with Con A, leukocytes cultured from outdoor animals produced a lower amount of IFNγ compared with those cultured from indoor animals at both summer and winter sampling points (P < 0.05, Figure 2). A season effect was also observed between summer and winter with a higher level of IFNγ production in both groups in winter (P < 0.05).

### Proliferation of PBMC

No statistical differences were revealed in the proliferation of PBMC isolated from indoor or outdoor animals upon Con A stimulation in summer or winter.

### Histology of the abomasal mucosa

Histological examination of the samples showed deep gastric pits above the glandular region. Mucosal lymphoid follicles were evident in both outdoor and indoor animals. A lesion of the abomasum was evident in one animal from the outdoor group.

**Figure 3:**
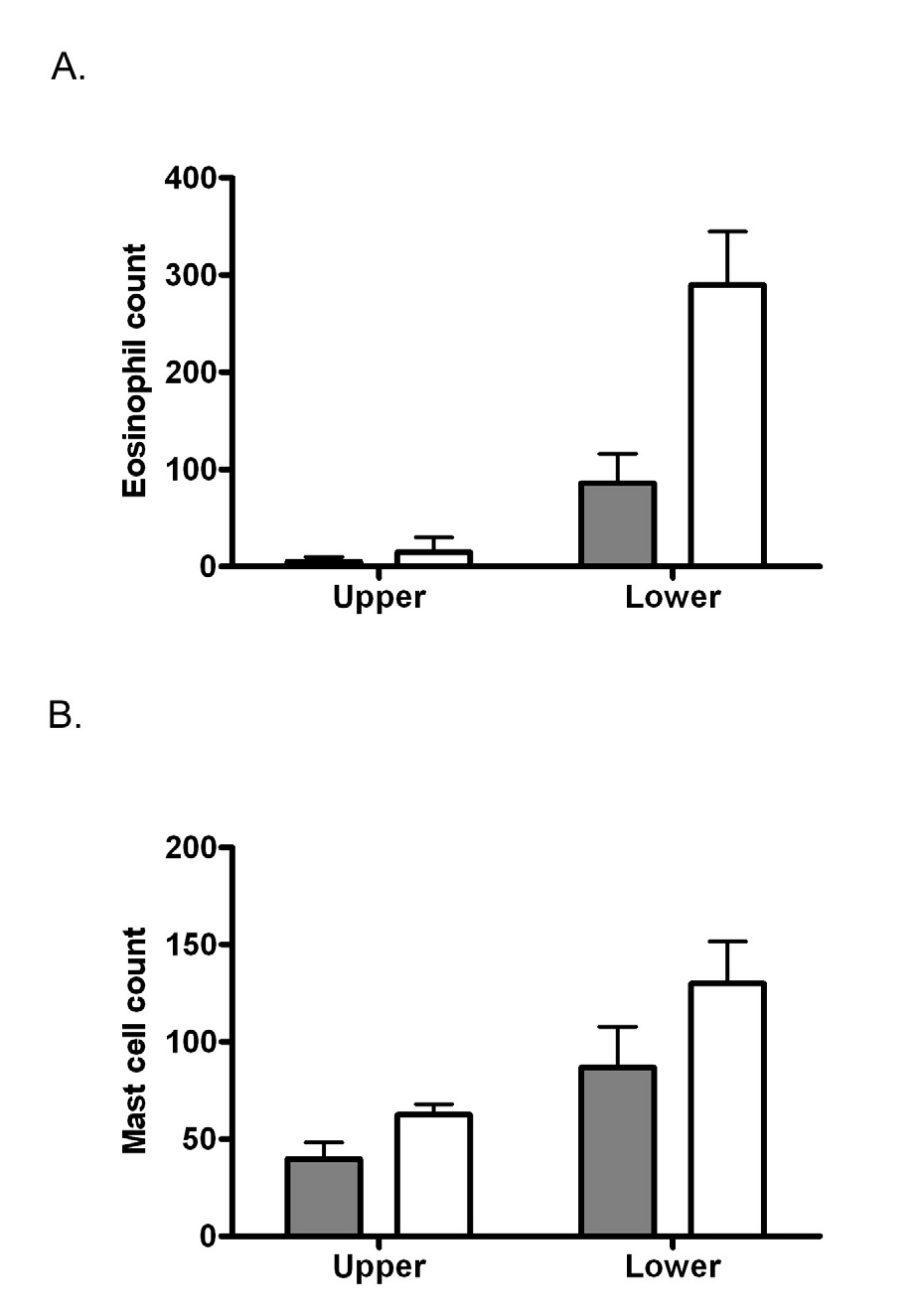
**Eosinophil and mast cell count in the abomasal mucosa**. Eosinophil (Figure 3A) and mast cell (Figure 3B) counts in the abomasal mucosa of indoor (shaded bars) and outdoor (clear bars) cattle during summer and winter are depicted as mean ± SEM. P values for the treatment, localization and treatment × localization effects are 0.020, 0.001, 0.006, respectively for eosinophils and 0.093, 0.001, 0.300, respectively for mast cells.

Eosinophils and mast cells were evident in the abomasal mucosa. In comparison to the upper mucosa, eosinophils were found in greatest numbers (P < 0.05) in the lower mucosa in both outdoor and indoor animals (Figure 3A). For both locations, the number of eosinophils was higher in outdoor than in indoor animals but the difference was greater in the lower mucosal regions resulting in an interaction between production system and location (P < 0.05). Mucosal mast cells in all sections were pleomorphic, ranging from small round cells to spindle-shaped cells with long processes. Again, mucosal mast cells were found in greatest numbers in the lower mucosa, in comparison to the upper mucosa, in both groups (P < 0.001, Figure 3B). The number of mast cells in both upper and lower mucosal regions was greater in outdoor animals than in indoor animals, although this only approached significance (P < 0.1).

### Cytokine gene expression in the small intestine

**Figure 4:**
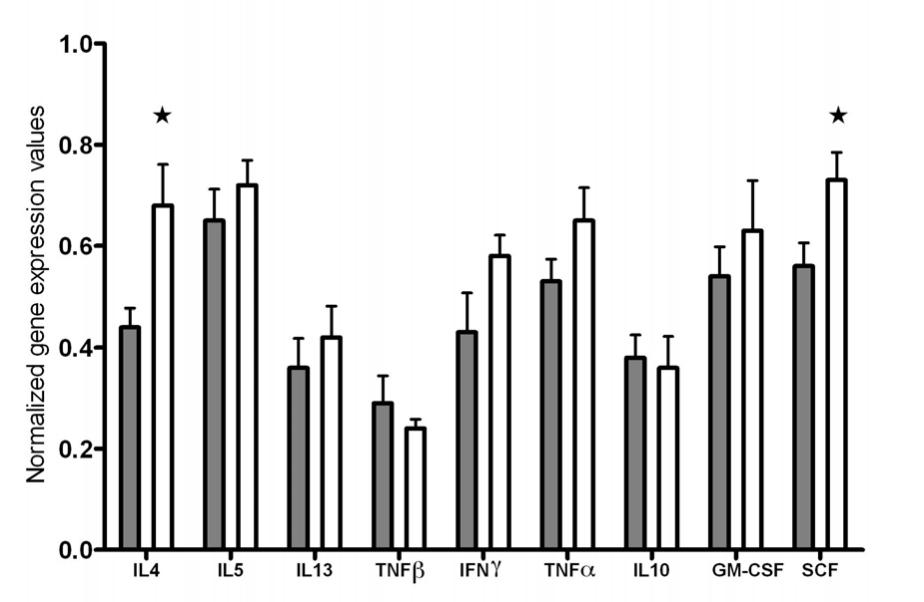
**Cytokine gene expression profile in the small intestine**. Cytokine gene expression profile in the small intestine of indoor (shaded bars) and outdoor (clear bars) animals at slaughter. Values are depicted as mean ± SEM. * P < 0.05.

Interleukin-4 and SCF were expressed at higher levels in outdoor animals compared to indoor animals (P < 0.05, Figure 4). Transcription levels of IL-5, IL-10, IL-13 as well as TNFα, TNFβ, IFNγ and GM-CSF were not significantly different between animals on the two production systems.

## Discussion

In this study, all the haematological values were within normal physiological ranges, and the animals in both production systems appeared to be clinically healthy throughout the study. Few studies have compared leukocyte counts between indoor and outdoor production systems. These have been limited to pigs and showed contrasting results; Kleinbeck and McGlone [4] reported a higher WBC count and a lower neutrophil percentage in indoor animals whereas Reed and McGlone [21] did not detect any change in cell numbers, similar to our results in cattle. Limited stall space allowance for housed animals has been shown to increase slightly the number of WBC, possibly due to stressful conditions [22]. Therefore, the lack of difference in leukocyte differential counts between the two production systems in the current study suggests that animals were managed in a non-stressful environment.

Erythrocyte numbers, hemoglobin and haematocrit measurements were all lower in outdoor cattle, in comparison to the indoor group, although all the values were within physiological ranges. As haematocrit is the proportion of blood volume that is occupied by red blood cells, a decrease in the number of these cells could lower the hematocrit. This could also lead to an overall decrease of hemoglobin concentration. The modification of the haematological parameters in this study could indicate a lack of iron, folic acid or vitamin B_12 _as these nutrients are known to decrease erythropoiesis [23]. Futhermore, copper limitation has been suggested to decrease iron utilization from diets causing a decrease of red blood cell parameters in cows [24]. As the level of copper was lower in outdoor animals, the relationship between iron, copper and haematological parameters should be further investigated. Interestingly, disorders that affect the gastrointestinal tract can impair iron absorption [25]. A higher pepsinogen concentration was found in the blood of outdoor cattle during summer and winter. The increase of serum pepsinogen concentration observed in the outdoor group is a well recognised sign of gastrointestinal helminth parasitism of animals raised on pasture. Consequently, the link between nematode infection and a possible reduction in iron uptake resulting in decrease of erythropoeisis in outdoor animals merits further attention.

To further characterize the immunity of both groups of animals, functional *in vitro *tests of immune cells were carried out. Since phagocytosis is an important factor in the defense of the host against all kinds of microorganisms, the slight reduction observed in the outdoor group could potentially have broad consequences for the immunity of these animals. The production of IFNγ, an important cytokine involved in Th1 cellular immunity, was assessed in this study and showed a reduction in the outdoor animals. As IFNγ is considered to be a pro-inflammatory cytokine, responsible for killing intracellular pathogens [26], this could possibly result in a less effective immune response of outdoor animals against these microorganisms. In this context, a comparison of the response to a harmful pathogen such as *Mycobacterium bovis *which elicits a strong Th1 response [27] could be of interest.

In our study, local immunity was also assessed at the level of the gastrointestinal tract. It is continuously challenged with foreign antigens from pathogens and food and constitutes an important barrier with the external environment. At the tissue level, the mucosal epithelium provides an environment in which commensal flora thrive, facilitating absorption of nutrients by the host, yet it defends the host against pathogenic organisms through innate and adaptative immune mechanisms. Serum pepsinogen was measured as a marker of the mucosal integrity. Abnormal pepsinogen concentrations revealed damage to this tissue and modification of its permeability caused by nematodes [28]. As previously discussed, blood from animals reared at pasture contained a greater level of pepsinogen. A correlation between serum pepsinogen concentrations and the extent of the larval infection has been reported before [29]. In our study, higher serum pepsinogen concentrations occurred in summer which is consistent with previously published data showing that the highest level of infestation is in July in temperate climates [29]. As expected, serum pepsinogen was lower in the indoor animals. Severe contamination by nematode larvae is unlikely since these animals were kept indoors for a year, and had been treated with anthelmintics at housing.

With regard to the abomasal data, recruitment of eosinophils and mast cells from the circulation into inflammatory foci has been documented in response to parasite, bacterial and viral infections [30,31]. In ruminants, numerous studies have reported an increase of these immune cells in the gastrointestinal tract in response to nematode infection [32-34]. The lack of increase in eosinophils in the blood together with the higher number in the abomasum mucosa suggests a displacement of the pool of these cells rather than an expansion of the total population. A similar mechanism has been previously suggested for plasma cells [35,36]. Another interesting finding of this study was the higher number of eosinophils in the glandular region of the mucosa. A similar numerical trend was also seen for mast cells.

Eosinophils and mast cells closely interact with each other through the production of a broad spectrum of inflammatory molecules such as cytokines, chemokines and growth factors [31,37]. Of the cytokine genes investigated in our study, only IL-4 and SCF were up-regulated in the small intestine. This up-regulation of IL-4 in the gastrointestinal tract of outdoor animals could contribute to the recruitment of eosinophils and mucosal mast cells [31,38,39]. Furthermore, stem cell factor (SCF) is also known to be involved in mast cell migration and, in conjunction with IL-4, mast cell growth and proliferation [40]. The classical response to nematode infection in mammals is a Th2-like response with a predominant IL-4 production [41]. In cattle, a more complex Th1/Th2-mixed cytokine pattern involving production of IFNγ and IL-4 has been reported during infection with *Ostertagia ostertagi *[42]. However, the Th1 response characterized by the production of IFNγ peaked early at 15 days post-infection and decreased thereafter whereas IL-4 was expressed for longer (43 days post infection). Together with our results, this could suggest a sustained expression of a limited number of cytokines, mainly IL-4, during long-term helminth infection in cattle.

The results identified a slight impact of the production system on the functionality of the peripheral immune system. Of significance was the fact that copper and iodine concentrations were lower in outdoor animals. This is consistent with a previous study [43]. The availability of minerals in the soil and feed has previously been studied in respect to their role in the function of the immune system [44]. For example, previously published results have demonstrated an impairment of phagocytic activity, mainly resulting in a decrease of bactericidal activity, in copper-deficient animals [45-48]. A wide range in blood copper concentrations have been reported for cattle. However, Underwood and Suttle [49] suggest that 3 to 9 micromol/L is an appropriate marginal limit. On this basis, grazing cattle sampled during the summer can be considered marginal for plasma copper status but deficient at the time of winter sampling. However there were no symptoms of clinical deficiency observed. A study of dairy cows also suggests that low dietary copper may decrease IFNγ production by mononuclear cells when stimulated with Con A [50]. Therefore, the lower level of copper in the outdoor reared cattle observed in this study could contribute to some extent to the functional differences in immune cells. Interestingly, the lower concentration of iodine in the outdoor animals could also contribute to some of the functional differences reported. Very low dietary iodine is a well known cause of hypothyroidism [51]. Interferon-γ production is reduced in immune cells isolated from lymph nodes of hypothyroidic mice [52].

## Conclusions

This study constitutes a first attempt to describe and compare the immunity of cattle raised in a long-term indoor versus long-term outdoor production system. Peripheral immune measurements did not reveal a strong difference between outdoor and indoor animals. Further work is needed to determine if the lower phagocytic activity and IFNγ production observed in outdoor cattle would result in a less efficient immune response when animals are challenged with pathogens. The potential implications of long-term low mineral status on the immune system, requires further investigation.

## Authors' contributions

AL, FM, AM, BE and TS designed the study and supervised the drafting and editing of the manuscript. AL undertook the immunological and gene expression work under the supervision of TS. AB and AM, managed the animal feeding trial. DC and FM undertook the sampling. TE performed the histological work under the supervision of DC. BE and PR assisted with immunological measurements. JOD provided input into the statistical analysis. All authors read and approved the final manuscript.
